# Does Checklist-Guided Shared Decision Making Have a Sustained Effect on Code Status Decisions Among Medical Inpatients? Long-Term Follow Up of the Randomized CLEAR Checklist Trial

**DOI:** 10.1007/s11606-026-10397-4

**Published:** 2026-04-14

**Authors:** Benjamin Bissmann, Samuel K. Zumbrunn, Sebastian Gross, Christoph Becker, Armon Arpagaus, Flavio Gössi, Leta Arpagaus, Rahel Kuster, Philipp Schuetz, Jörg D. Leuppi, Drahomir Aujesky, Balthasar L. Hug, Thomas Peters, Arnoud J. Templeton, Stefano Bassetti, Sabina Hunziker

**Affiliations:** 1 Medical Communication and Psychosomatic Medicine, University Hospital Basel, Basel, Switzerland; 2 Medical Outpatient Department, University Hospital Basel, Basel, Switzerland; 3 Faculty of Medicine, University of Basel, Basel, Switzerland; 4 Division of Internal Medicine, Cantonal Hospital Aarau, Aarau, Switzerland; 5 University Institute of Internal Medicine, Cantonal Hospital Baselland, Liestal, Switzerland; 6 Department of General Internal Medicine, Inselspital, Bern University Hospital, University of Bern, Bern, Switzerland; 7 Division of Internal Medicine, Cantonal Hospital Lucerne, Lucerne, Switzerland; 8 Division of Internal Medicine, St. Claraspital, Basel, Switzerland; 9 Department of Medical Oncology, St. Claraspital, Basel, Switzerland; 10 St. Clara Research, Basel, Switzerland; 11 Division of Internal Medicine, University Hospital Basel, Basel, Switzerland; 12 Faculty of Health Sciences and Medicine, University of Lucerne, Lucerne, Switzerland

**Keywords:** code status discussion, shared decision-making, cardiopulmonary resuscitation, advanced directive

## Abstract

**Background:**

Code status discussions are often inadequate, omitting key information on prognosis and resuscitation risks. In the randomized controlled CLEAR trial, use of a checklist to guide shared decision-making for code status discussions improved the quality of care by reducing patients’ uncertainty and increasing their satisfaction with discussions and knowledge of this topic. Moreover, a higher rate of “do-not-resuscitate” (DNR) code status preference was observed during the index hospital stay. The present analysis evaluates whether these effects were sustained over time.

**Objective:**

To evaluate the documented code status (primary outcome) and exploratory secondary outcomes, including patients’ general knowledge about resuscitation measures after a mean follow-up time of 3.2 years, assessed by telephone interview.

**Design:**

Long-term follow-up of a prospective, randomized controlled study.

**Participants:**

Of the 1954 medical inpatients in the previous CLEAR trial, 604 had died and 10 were excluded due to missing information, leaving 1340 patients for analysis (mean age 65.0 (SD ± 16.3) years, 44.1% female).

**Interventions:**

Code status discussions using a shared decision-making checklist including visual decision support addressing prognosis, resuscitation outcomes, and patient values compared to usual care.

**Main Measures:**

Last documented code status at follow-up.

**Key Results:**

Patients in the intervention group had a significantly higher rate of documented DNR status at follow-up compared to patients in the usual care group (251 [36.1%] vs. 191 [29.8%]; adjusted risk ratio 1.23, 95% CI 1.04 to 1.45, *p* = 0.02). No significant differences were observed in knowledge about resuscitation measures.

**Conclusions:**

Several years after a shared decision-making code status discussion, patients recalled little specific knowledge about resuscitation measures and their prognostic outcomes. However, the intervention group maintained a higher documented preference for DNR. These results highlight the need for repeated discussions, including prognosis and expected outcomes, to support informed and consistent decision-making.

## INTRODUCTION

In-hospital cardiac arrest (IHCA) is a sudden and severe event.^1^ With average incidences of 1.5 to 2.8 per 1000 hospital admissions in Europe and up to 10 per 1000 hospital admissions in the USA, respectively, IHCA remains a relatively frequent and relevant incident.^1,2^ IHCA carries a poor prognosis, with average survival rates to hospital discharge ranging from 15 to 34%.^1–4^ Among survivors, up to 40% experience significant neurological deficits.^5,6^ Considering the sudden and often unpredictable onset of cardiac arrest, it is essential that hospital clinicians consistently engage in and document discussions regarding patients’ preferences for advanced life support measures.^7–9^ Previous research indicates that code status discussions are often inadequate, as essential information about resuscitation risks, prognosis, and potential adverse effects is frequently omitted.^10,11^ Furthermore, the general public tends to overestimate post-cardiac arrest survival, despite prognosis being a key determinant of code status preference.^12^

To fill this gap, a checklist-guided approach was developed in the randomized controlled CLEAR (***C****linician-Patient Engagement*, ***L****earn and Inform*, ***E****xplore Patient Preferences*, ***A****ssess and Document*, ***R****eview Advance Directives*) trial. This trial, conducted between June 2019 and April 2023 at six internal medicine wards across Switzerland, valuated a structured communication intervention aimed at improving the quality of code status discussions. The intervention consisted of a brief physician communication training supported by a standardized checklist and visual decision aid to facilitate shared decision-making, including discussion of prognosis, resuscitation outcomes, and patient values. In the original trial, use of the checklist-guided shared decision-making (SDM) approach resulted in several improved quality of care measures, including reduced uncertainty regarding code status decisions, better involvement in the decision-making process, and increased satisfaction with resuscitation discussions and increased knowledge about resuscitation and its outcomes among patients and families. Additionally, a 37% higher preference for a “do-not-resuscitate” (DNR) code status was observed during the hospital stay.^13^ The intervention combined a 1-h physician communication training with the use of a structured SDM checklist and visual decision aid to support clear information exchange, exploration of patient values, and documentation of preferences. These findings, which are in line with other smaller trials that similarly demonstrated improved communication quality and more patient-centered code status decisions,^14^ have important implications for short-term clinical application, especially as communication interventions such as decision aids and SDM have consistently been shown to improve the overall quality of care.^15,16^ However, it remains unclear whether these positive effects persist over time and how much of the provided information patients are able to recall about this topic several years after hospital discharge. To our knowledge, there is no conclusive research regarding the long-term effects of specific communication interventions on code status discussions.

In this follow-up analysis, we therefore aim to evaluate the long-term effects of checklist-guided SDM with decision aids on the sustainability in patients’ code status preferences, assessed by their last documented code status. In a further step, we aim to assess its potential impact on rehospitalization rates, ICU admissions, resuscitation outcomes, and the completion of advance directives.

## METHODS

### Study Setting

This is an ancillary project to the CLEAR trial^13^ looking at the impact of a standardized checklist-guided SDM communication intervention supported by decision aids on patients’ code status preferences, knowledge and satisfaction. In brief, the initial study was a pragmatic, cluster-randomized, multicenter trial conducted in the general internal medicine departments of six Swiss teaching hospitals (University Hospital Basel, Cantonal Hospital Aarau and Cantonal Hospital Basel-Land, Inselspital/Bern University Hospital, Cantonal Hospital Lucerne, and St. Claraspital Basel) between June 2019 and April 2023. The study was registered at ClinicalTrials.gov (https://clinicaltrials.gov/ct2/show/NCT03872154). This ancillary project was approved by the local Ethics Committee (Northwest and Central Switzerland as well as Ethics Committee Bern, project ID EKNZ 2019-00078) on 14 June 2024.

### Study Design and Participants

**Figure 1:**
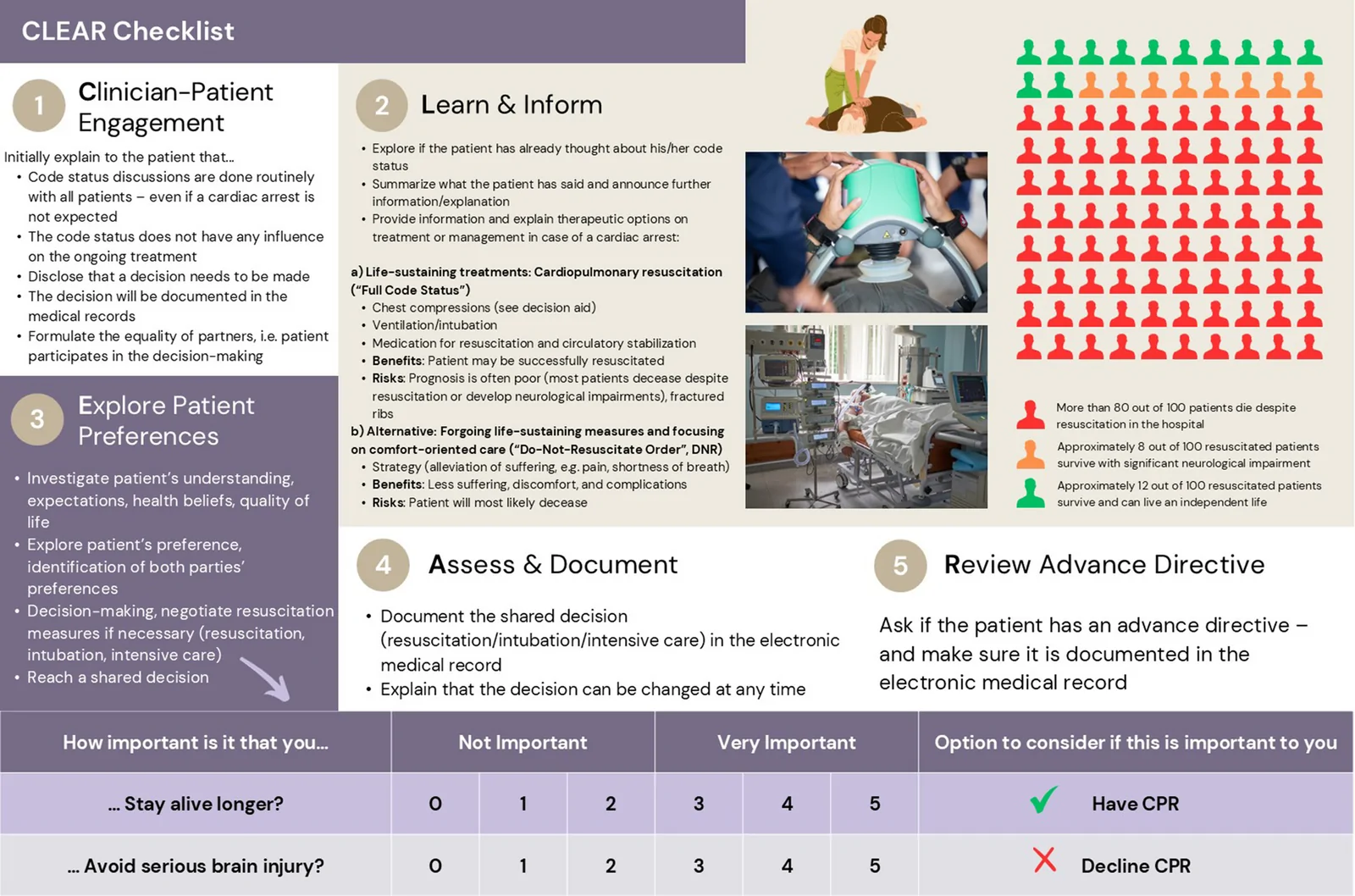
CLEAR—Checklist and decision aid used in the original trial

In the CLEAR trial, residents (cluster) were randomly allocated to either an intervention group or a control group. The CLEAR trial is briefly summarized here in the context of study implementation; detailed outcome definitions are provided in the “Outcome Measures” section below. Residents in the checklist intervention group attended a 1-h communication workshop conducted by members of the research team, all experienced in teaching medical communication. The session covered principles of SDM, prognostic factors, and expected outcomes of in-hospital resuscitation. Thus, the intervention represented a structured communication training program supported by decision aids, rather than an educational or informational handout alone. The training emphasized how to engage patients in shared decision-making, guide conversations about prognosis, and document preferences using the CLEAR checklist and decision aids. Participants were trained to use a checklist and decision aids (Fig. 1) incorporating images of a patient undergoing CPR, a mechanically ventilated patient in the ICU, and a graph depicting realistic CPR outcomes. The decision aid was based on published data and partially adapted from tools in the Ottawa Decision Aid Inventory.^17^

The checklist was developed in line with SDM research and guidelines,^18^ condensed into the acronym CLEAR. During the workshop, residents practiced simulated code status discussions using these tools, followed by targeted instructor feedback on their application in clinical contexts. To reduce bias, residents in the usual care group participated in a separate 1-h communication workshop focused on the importance of code status discussions and general communication techniques, such as organizing information and addressing emotions. This session did not include information on the checklists and the decision aids. Each resident enrolled 10–15 consecutive patients. The checklist was applied during code status discussions conducted over a period of approximately 3 weeks and was used once per patient.

To evaluate medical futility, the Good Outcome Following Attempted Resuscitation (GO-FAR) score^19^ and the Clinical Frailty Scale (CFS)^20,21^ were used during the screening process. Patients were considered to have a futile prognosis in case of a cardiac arrest, and excluded from the SDM intervention if they scored ≥ 14 on the GO-FAR or ≥ 7 on the CFS. These patients were enrolled in a separate trial (GUIDE Trial).^22^ After the clinical code status discussion had taken place, informed consent for study participation was obtained prior to the blinded patient interview by a study team member to assess secondary outcomes. Details of randomization, blinding, eligibility criteria, and communication training have been outlined in previous publications.^13^

For this preplanned follow-up study, all patients who had provided informed consent during the original CLEAR trial, including consent for future recontact, were eligible for inclusion. Medical records and published obituaries were screened to identify eligible patients. Survivors were contacted by telephone for a follow-up interview using a questionnaire based on the one used in the original CLEAR trial and pretested with 10 hospitalized patients. Patients were excluded if they were unreachable after three attempts, cognitively impaired as determined by review of the electronic medical records for diagnoses indicating cognitive impairment or dementia, had no contact information, or had died since the primary study. In accordance with the approved protocol, oral informed consent was reconfirmed from participants before conducting the follow-up telephone interviews. Details regarding the procedures for telephone contact and data abstraction are described in more detail below.

### Data Collection

Baseline data were collected before the initial code status discussion and included patients’ sociodemographic information, primary diagnosis, and comorbidities associated with their hospitalization. The CFS^20,21^ and the GO-FAR score^19^ were assessed for each patient by a study team member. Medical records were reviewed to calculate the Charlson Comorbidity Index (CCI)^23^ and the National Early Warning Score (NEWS).^24^ Long-term follow-up was conducted between June and October 2024. This involved reviewing medical records and published obituaries to gather information on patients’ vital status, rehospitalizations, new medical diagnoses, and changes in code status. For each patient, the follow-up data were abstracted by one author with a preliminary defined online abstraction form. From the medical records, we collected data for the primary outcomes as well as some secondary outcomes like patients’ code status changes since the main trial, number of planned and unplanned hospitalizations, and number of ICU admissions. For the remaining secondary outcomes, we conducted a telephone interview.

### Outcome Measures

The primary outcome was the patients’ code status documented in the electronic medical records of their most recent hospitalization, consisting of DNR code status, preference for ICU admission, and preference for mechanical ventilation. All secondary outcomes were exploratory and included patients’ code status changes since the main trial, number of planned and unplanned hospitalizations, number of ICU admissions, completion of advanced directives, recollection of initial code status discussion in main study, the CFS determined through oral interview and its progression, self-reported previous discussion of personal code status with relatives, the preference for physician recommendation regarding code status decision, and patients’ general knowledge about resuscitation measures. Patients’ knowledge about resuscitation measures was assessed using a validated six-item questionnaire measuring general understanding of CPR procedures, survival probabilities, and neurological outcomes after in-hospital cardiac arrest (score range 0–6, with higher scores indicating greater knowledge). This measure reflects general awareness of CPR and its prognosis, not individualized patient-specific education.^25,26^ Details of outcomes are presented in Table 2.

### Statistical Analysis

We used a similar statistical approach as in the initial CLEAR trial. In brief, to describe the patient cohort, continuous variables were summarized as means (± standard deviation, SD), while categorical or binary variables were reported as frequencies and proportions (percentages). For secondary outcomes, effect estimates with 95% confidence intervals are reported without *p*-values, as these analyses were exploratory. Pearson’s *X*^2^-test was used to compare binary and categorical variables. Continuous data was checked graphically for normality of the distribution and compared using the *t*-test as appropriate. Cumulative incidence of outcomes at the individual level was compared with the use of a binomial regression model with robust sandwich standard errors to account for grouping within clusters (residents). The intra-cluster correlation coefficient (ICC) was 0.062 (95% CI 0.026 to 0.14). A generalized linear model with a binomial distribution and a log-link function was defined to estimate the risk ratio as a measure of effect. The analyses were adjusted for the baseline variables of patient age, sex, principal admission diagnosis, and study center as outlined in the underlining study protocol of the main trial. All statistical analyses were conducted using Stata V.15.0 (StataCorp, College Station, TX, USA) for all statistical analyses. A *p*-value of < 0.05 was considered statistically significant.

## RESULTS

### Study Population

**Figure 2:**
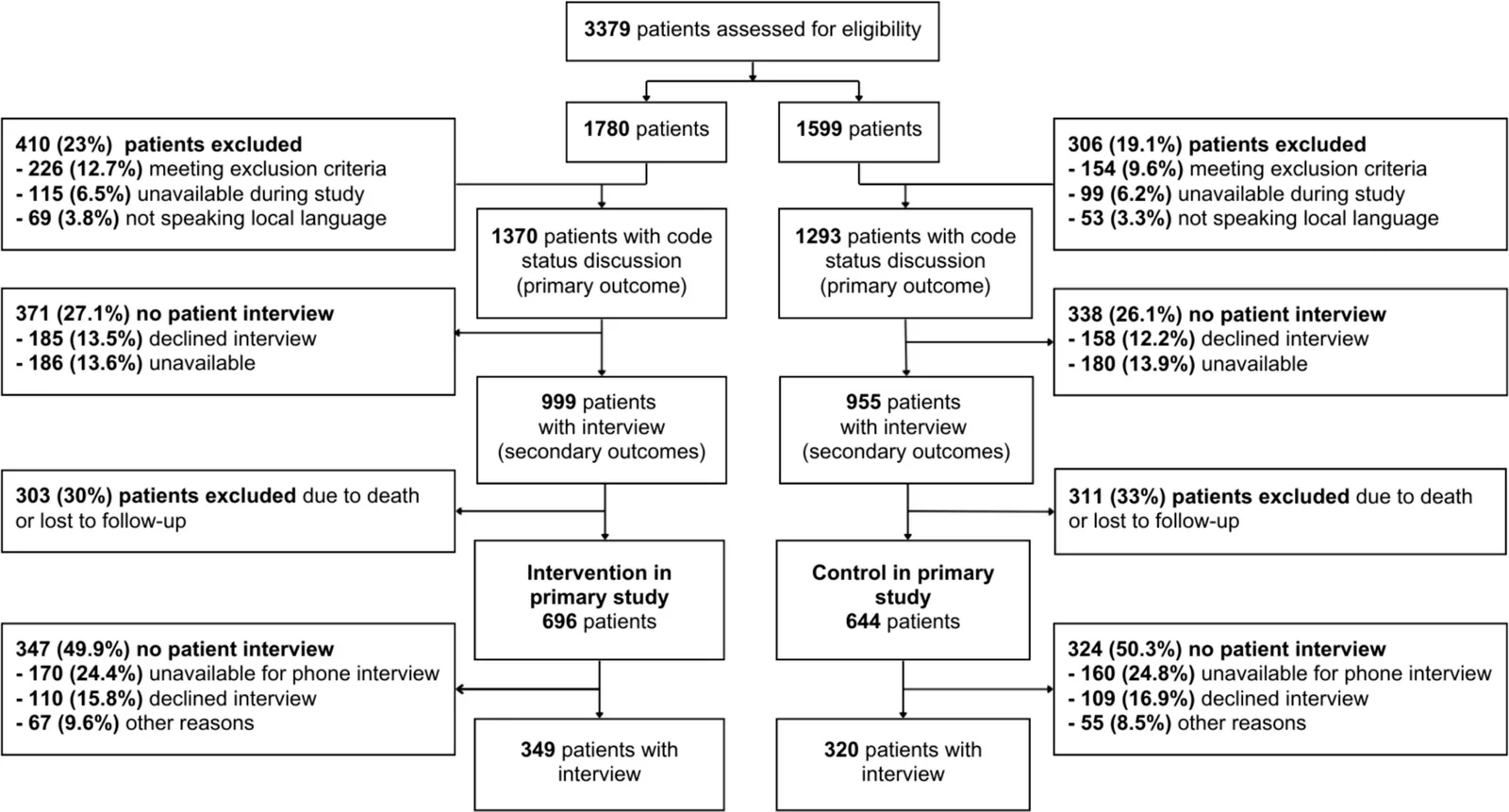
Flowchart

A detailed study flowchart is shown in Fig. 2. Among the 1954 patients who participated in the interviews during the CLEAR trial, 604 (30.9%) had died by the time of follow-up and 10 (0.5%) were lost to follow-up, leaving 1340 patients eligible for analysis of the primary outcome, including 696 (51.9%) in the intervention group and 644 (48.1%) in the control group. A subset of 669 patients (349 in the intervention group and 320 in the control group) were successfully contacted and completed the telephone interview, providing self-reported data on secondary outcomes such as recollection of the initial discussion, current preferences, and knowledge of resuscitation measures. The mean time to follow-up was 3.2 years (SD ± 0.9) years.

Baseline characteristics of enrolled patients stratified among randomization arms are shown in Table 1. The mean age was 65.0 years (SD ± 16.3 years), 591 (44.1%) were female, and 840 (62.7%) patients have had at least one hospital readmission since the main study. Baseline characteristics were well balanced between groups.

**Table 1:** Baseline Characteristics

Factor	Sample size	All	Usual care group	Checklist-group	*p*-value
Patients (*n*)		1340	644	696	
Sociodemographic factors at baseline (CLEAR trial)					
Age (years), mean (SD)	1340	65.0 (16.3)	64.8 (16.5)	65.1 (16.0)	0.74
Female gender, *n* (%)	1340	591 (44.1%)	299 (46.4%)	292 (42.0%)	0.099
Swiss citizenship, *n* (%)	1340	1079 (80.5%)	518 (80.4%)	561 (80.6%)	0.94
Religious affiliation, *n* (%)	1339	948 (70.8%)	456 (70.8%)	492 (70.8%)	0.99
*Job situation, n (%)*	1339				
Working/in education		411 (30.7%)	196 (30.5%)	215 (30.9%)	0.92
Unemployed		33 (2.5%)	17 (2.6%)	16 (2.3%)	
Retired/disability pension		859 (64.2%)	411 (63.9%)	448 (64.4%)	
Other		36 (2.7%)	19 (3.0%)	17 (2.4%)	
Health related factors					
*Main diagnoses at baseline (CLEAR trial)*	1340				
Ischemic heart disease, *n* (%)		142 (10.6%)	65 (10.1%)	77 (11.1%)	0.41
Heart failure, *n* (%)		90 (6.7%)	47 (7.3%)	43 (6.2%)	
Other cardiologic diseases, *n* (%)		156 (11.6%)	67 (10.4%)	89 (12.8%)	
Tumor, *n* (%)		114 (8.5%)	46 (7.1%)	68 (9.8%)	
Infectious diseases, *n* (%)		160 (11.9%)	83 (12.9%)	77 (11.1%)	
Respiratory diseases, *n* (%)		78 (5.8%)	41 (6.4%)	37 (5.3%)	
Gastroenterological diseases, *n* (%)		156 (11.6%)	74 (11.5%)	82 (11.8%)	
Metabolic diseases, *n* (%)		64 (4.8%)	33 (5.1%)	31 (4.5%)	
Neurological diseases, *n* (%)		105 (7.8%)	46 (7.1%)	59 (8.5%)	
Other, *n* (%)		275 (20.5%)	142 (22.0%)	133 (19.1%)	
*Main diagnoses leading to first re-hospitalization*	820				
Ischemic heart disease, *n* (%)		58 (7.1%)	29 (7.1%)	29 (7.0%)	0.26
Heart failure, *n* (%)		47 (5.7%)	21 (5.2%)	26 (6.3%)	
Other cardiologic diseases, *n* (%)		58 (7.1%)	27 (6.6%)	31 (7.5%)	
Malignant tumor, *n* (%)		95 (11.6%)	46 (11.3%)	49 (11.9%)	
Infectious diseases, *n* (%)		95 (11.6%)	54 (13.3%)	41 (9.9%)	
Respiratory diseases, *n* (%)		27 (3.3%)	16 (3.9%)	11 (2.7%)	
Gastroenterological diseases, *n* (%)		102 (12.4%)	43 (10.6%)	59 (14.3%)	
Metabolic diseases, *n* (%)		29 (3.5%)	11 (2.7%)	18 (4.4%)	
Neurological diseases, *n* (%)		42 (5.1%)	27 (6.6%)	15 (3.6%)	
Other, *n* (%)		267 (32.6%)	133 (32.7%)	134 (32.4%)	
*Comorbidities at follow-up*					
New or progressive malignant diagnosis, *n* (%)	1340	93 (6.9%)	49 (7.6%)	44 (6.3%)	0.35
Thereof metastasized, *n* (%)	93	21 (23%)	9 (18%)	12 (27%)	0.31
Progression of the non-malignant underlying disease, *n* (%)	1340	165 (12.3%)	75 (11.6%)	90 (12.9%)	0.47
*Clinical values*					
Charlson Comorbidity Index at baseline, mean (SD)	1340	3.8 (2.5)	3.8 (2.4)	3.9 (2.5)	0.83
Charlson Comorbidity Index at follow-up, mean (SD)	1340	4.8 (3.0)	4.9 (3.0)	4.8 (3.1)	0.49
Clinical Frailty Scale at baseline, mean (SD)	1340	3.3 (1.2)	3.3 (1.2)	3.2 (1.2)	0.093
NEWS2-Score at baseline, mean (SD)	1340	1.7 (1.9)	1.7 (2.0)	1.8 (1.9)	0.67
GO-FAR Score at baseline, mean (SD)	1340	−3.6 (5.8)	−3.7 (5.9)	−3.6 (5.7)	0.73
Survival according to GO-FAR Score at baseline, *n* (%)	1340				
27% chance of survival		611 (45.6%)	300 (46.6%)	311 (44.7%)	0.49
9.4% chance of survival		729 (54.4%)	344 (53.4%)	385 (55.3%)	

*n* number, *SD* standard deviation, *NEWS2* National Early Warning Score 2, *GO-FAR* Good Outcome Following Attempted Resuscitation

### Primary Outcome: Last Documented Code Status Preference

**Figure 3:**
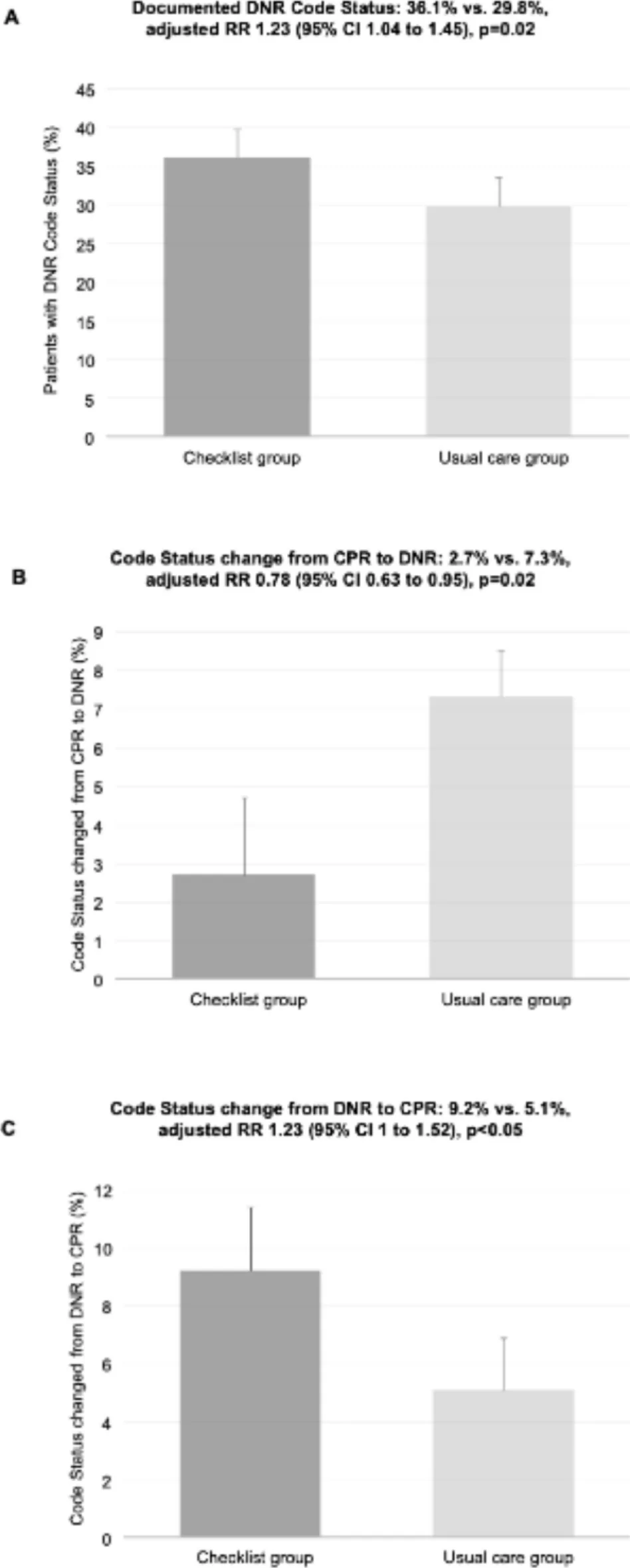
Frequency of code status and changes at follow-up. A The distribution of documented code status at long-term follow-up for both groups. B, C Transitions of code status from baseline to follow-up (from full code to DNR and from DNR to full code, respectively), allowing a direct visual comparison of the magnitude and direction of changes between groups

Regarding the primary outcome, patients in the intervention group had a significantly higher rate of documented DNR status at follow-up compared to patients in the usual care group (251 [36.1%] vs. 191 [29.8%]; adjusted risk ratio 1.23, 95% CI 1.04 to 1.45, *p* = 0.02). There was no difference regarding the documented preference for intensive care unit admission (596 [87.6%] vs. 545 [87.2%]; adjusted risk ratio 1.02, 95% CI 0.98 to 1.06, *p* = 0.37) and the documented preference for mechanical ventilation in case of clinical deterioration (565 [83.7%] vs. 515 [82.9%]; adjusted risk ratio 1.02, 95% CI 0.96 to 1.08, *p* = 0.53) (Table 2, Fig. 3A).

**Table 2:** Primary and Secondary Outcomes

Factor	Sample size	All (*n* = 1340)	Usual care group (*n* = 644)	Checklist-group (*n* = 696)	*p*-value	Adjusted RR or difference (95% CI)*	*p*-value	Adjusted RR or difference (95% CI)**	*p*-value
Primary outcome									
*Last documented code status from medical record*									
Documented DNR code status, n (%)	1340	443 (33.1%)	191 (29.8%)	251 (36.1%)	0.02	1.23 (1.04, 1.44)	0.01	1.23 (1.04, 1.45)	0.02
Documented preference for ICU admission, *n* (%)	1305	1141 (87.4%)	545 (87.2%)	596 (87.6%)	0.81	1.02 (0.97, 1.08)	0.43	1.02 (0.98, 1.06)	0.37
Documented preference for mechanical ventilation, *n* (%)	1296	1080 (83.3%)	515 (82.9%)	565 (83.7%)	0.71	1.02 (0.96, 1.09)	0.5	1.02 (0.96, 1.08)	0.53
Secondary outcomes									
*Code status sustainability*									
Code status changed since main study, *n* (%)	1340	163 (12.2%)	80 (12.4%)	83 (11.9%)		1.02 (0.84, 1.25)		1 (0.81, 1.22)	
Code status changed from CPR to DNR, *n* (%)	1340	66 (4.9%)	47 (7.3%)	19 (2.7%)		0.79 (0.66, 0.95)		0.78 (0.63, 0.95)	
* Code status changed from DNR to CPR, n (%)*	1340	*97 (7.2%)*	*33 (5.1%)*	*64 (9.2%)*		*1.28 (1.05, 1.55)*		*1.23 (1, 1.52)*	
*Hospital readmissions*									
Hospital readmission, *n* (%)	1340	840 (62.7%)	415 (64.4%)	425 (61.1%)		0.98 (0.88, 1.09)		0.96 (0.87, 1.07)	
Number of hospital readmissions, mean (SD)	1340	2.0 (3.3)	2.1 (3.1)	1.8 (3.4)		−0.27 (−0.64, 0.1)		−0.26 (−0.6, 0.07)	
ICU admission, *n* (%)	1340	168 (20.3%)	88 (21.5%)	80 (19.1%)		0.85 (0.7, 1.04)		0.87 (0.72, 1.05)	
*Patient’s recollection of initial code status discussion in main study*									
Patient’s recollection of initial code status discussion in main study, *n* (%)	669	233 (34.8%)	104 (32.5%)	129 (37.0%)		1.05 (0.85, 1.29)		1.06 (0.86, 1.31)	
*Clinical Frailty Scale*									
Clinical Frailty Scale at follow-up, orally assessed, mean (SD)	650	3.1 (1.5)	3.2 (1.5)	3.1 (1.5)		−0.16 (−0.41, 0.1)		−0.15 (−0.38, 0.08)	
Worsening of Clinical Frailty Scale, *n* (%)	650	259 (39.8%)	113 (36.6%)	146 (42.8%)		1.15 (0.94, 1.4)		1.1 (0.91, 1.33)	
*Completion of advanced directives*									
Completion of advanced directives at all timepoints, *n* (%)	651	329 (50.5%)	159 (50.8%)	170 (50.3%)		0.92 (0.78, 1.08)		0.89 (0.76, 1.05)	
*Involvement of relatives*									
Self-reported previous discussion of personal code status with relatives, *n* (%)	663	508 (76.6%)	241 (76.5%)	267 (76.7%)		0.97 (0.88, 1.07)		0.96 (0.87, 1.05)	
*Reported patient’s current preferences regarding code status discussion*									
Preference for physician recommendation regarding code status decision, yes, *n* (%)	622	461 (74.1%)	216 (73.7%)	245 (74.5%)		0.94 (0.84, 1.06)		0.95 (0.85, 1.05)	
*Patient's knowledge about resuscitation*									
Patients’ knowledge about resuscitation (0–6), mean (SD)	644	2.5 (1.1)	2.5 (1.1)	2.5 (1.1)		0.05 (−0.13, 0.24)		0.03 (−0.13, 0.2)	

*Adjusted for cluster, **Adjusted for cluster, diagnosis at baseline, age, gender, center; Abbreviations: *RR* risk ratio, *SD* standard deviation, *CI* confidence interval, *SD* standard deviation, *n* number, *DNR* do-not-resuscitate, *CPR* cardiopulmonary resuscitation, *ICU* intensive care unit, *VAS* visual analogue scale

### Secondary Outcomes

#### Code Status Sustainability

There were similar proportions of patients in both study groups changing their code status during follow-up, i.e., 83/696 [11.9%] in the intervention group vs. 80/644 [12.4%] in the control group (adjusted risk ratio 1.0, 95% CI 0.81 to 1.22). However, among those patients changing the code status, in the intervention group, there were less patients changing from full code status to DNR compared to the usual care group (19/696 [2.7%] vs. 47/644 [7.3%]; adjusted risk ratio 0.78, 95% CI 0.63 to 0.95). On the other hand, the change from DNR to full code status was more often observed in the intervention group compared to the usual care group (64/696 [9.2%] vs. 33/644 [5.1%]) (Table 2, Fig. 3B and C).

#### Patients’ Knowledge About Resuscitation Measures

There was no significant difference between the groups regarding the patient’s knowledge about resuscitation measures, which was assessed using a validated six-item knowledge score (range 0–6, with higher scores indicating greater knowledge) (mean score 2.5 [SD ± 1.1] vs. 2.5 [SD ± 1.1]; adjusted difference 0.03, 95% CI −0.13 to 0.2) (Table 2).

#### Additional Outcomes

All other outcomes such as number of hospital readmissions, ICU admissions, initial recollection of the code status discussion in the main study, the orally assessed CFS and its worsening since the main study, the completion of an advance directive at all timepoints, the self-reported previous discussion of personal code status with relatives, and the preference for physician recommendation regarding code status decision were comparable across randomization groups with no significant difference (Table 2).

## DISCUSSION

This long-term follow-up of the multicenter CLEAR trial aimed to evaluate whether the effects of checklist-guided shared decision-making on patients’ code status decisions (primary outcome) and other secondary outcomes persisted over time. While patients in the intervention group retained a significantly higher documented preference for DNR orders, several years post-intervention, both groups showed a notable decline in specific knowledge about resuscitation measures and their associated prognostic outcomes over time. These findings emphasize the need for sustained engagement and repeated discussion with patients regarding code status and information about resuscitation measures. Repeated conversations are critical, as they may help reinforce patients’ understanding of resuscitation options and ensure their preferences remain informed and aligned with their current health status and values.

Interestingly, only about 12% of patients changed their initial code status during follow-up. In the intervention group, fewer patients changed from full code status to DNR, while more patients changed from DNR to full code status compared to the control group. This bidirectional change may reflect the influence of SDM practices, particularly in empowering patients to reevaluate their preferences in response to changing health statuses or personal values. At the same time, alternative interpretations should be considered. Some initial DNR preferences may have been situational or transient, reflecting acute illness or emotional distress during hospitalization, and later reconsidered as patients’ clinical status or perspectives changed. The higher frequency of changes from DNR to CPR in the intervention group also highlights that early decisions should not be viewed as fixed, and that preferences may evolve over time. As this study was not designed to assess the appropriateness of individual decisions, these findings should be interpreted cautiously and primarily underscore the dynamic nature of code status preferences.

Nevertheless, the initial preference for DNR in the intervention group was still observed but less pronounced compared to the initial trial (50% in the initial trial vs. 36% in the follow-up), suggesting that while Checklist-guided SDM code status discussions have a lasting impact, the influence may wane over extended periods without reinforcement. The diminished effect likely reflects that, during rehospitalizations, code status discussions may have reverted to usual care rather than the initial SDM approach, as the physicians involved at that time were not study participants and had not received the specific SDM training.

Although the checklist and accompanying discussions addressed related treatment topics such as intubation, ventilation, and circulatory stabilization, preferences regarding ICU admission and mechanical ventilation remained largely unchanged in both groups. This finding suggests that the intervention may have predominantly influenced resuscitation-related decisions rather than broader treatment preferences. Further research would be needed to understand how patients integrate these specific treatment aspects into their overall goals of care.

An important finding of this follow-up was that long-term knowledge about resuscitation measures and prognostic implications diminished over time in all patients compared to the initial evaluation, with no more differences among groups (4.0 ± 1.5 points in the intervention group vs. 3.2 ± 1.5 points in the control group in the main trial vs. 2.5 ± 1.1 in both groups in the follow-up). This aligns with prior research, such as Saigal et al. who found significant patient recall decline for medical risks over time and showed that patients could recall only 18% of discussed risk about spinal surgery after 1 year.^27^ Yet, the lack of sustained differences in CPR-related knowledge at follow-up should be viewed in the context of the intervention’s primary aim, which was to support individualized decision-making rather than to ensure long-term retention of factual information. This interpretation is further reinforced by the well-known limitations of long-term recall of medical risk information. Also, it is possible that the initial intervention may have produced durable effects on values formation and the patient–physician relationship—thus maintaining preferences even in the absence of preserved cognitive knowledge. Overall, this highlights the transient nature of patient understanding and the need for routine reinforcement. For hospitalized patients, especially older adults, multimorbid individuals, this emphasizes the importance of readdressing code status preferences at each hospital admission, a practice supported by existing guidelines for advance care planning.^1,2^ The relatively common occurrence of IHCA in these populations further supports the necessity of ongoing discussions to ensure informed, contextually appropriate decisions.

Importantly, this study adds to the growing evidence supporting SDM approaches in clinical practice. As demonstrated in the original CLEAR trial, a SDM approach with decision aids including information about potential outcomes, prognosis, and resuscitation measures improves the quality of decision-making by strengthening patients’ confidence in their decisions, enhancing their involvement, and increasing their knowledge and satisfaction.^13^ Our study underlines the critical role of open communication, as patients frequently express the need for transparency, honesty, and detailed explanations about survival chances and the risks associated with resuscitation. This reflects patients’ desire for meaningful participation in their healthcare decisions, which aligns with the principles of SDM. By addressing these specific needs, clinicians can foster trust and ensure that patients feel heard and understood, ultimately leading to more patient-centered care. Moreover, the long-term benefits of the checklist-guided code status discussion are particularly notable. The intervention group in the CLEAR trial demonstrated a sustained increase in DNR preferences over time, suggesting that well-informed and collaborative discussions lead to less active management in case of a cardiac event. This is especially critical in the context of an aging population with an increasing burden of multimorbidity, where hospital readmissions and IHCAs are more common.^28,29^ Proactively incorporating SDM approaches may potentially mitigate these risks by aligning care plans with patients’ prognoses, goals, and wishes, ultimately improving overall healthcare outcomes.

The observed decline in patients’ knowledge and recall about resuscitation measures and prognostic outcomes several years after hospitalization may represent an important barrier to sustaining informed and consistent decision-making over time. The difficulty for patients in making a medical decision far in advance of a potential clinical deterioration remains a challenge.^30–32^ Many patients overestimate their survival chances following cardiac arrest, underscoring the need for continued education about prognosis and outcomes.^12^ Repeated discussions, complemented by decision aids, could help address this knowledge gap and support patients in making more informed and stable code status decisions.

The strength of this study includes the large pragmatic, cluster-randomized and multicenter study, which ensures a high internal and external validity. However, we are aware of several limitations: First, there was selection bias regarding secondary outcomes because not all patients participated in the telephone interviews (e.g., we excluded patients who were unreachable, had cognitive impairment, or died during follow-up). Additionally, there was a high number of patients who died during follow-up. Second, we did not evaluate whether end-of-life care or care during rehospitalizations was consistent with patients’ documented code status preferences. Although such information would provide important insights into the overall clinical impact of the intervention, these data were not consistently available across sites and were beyond the predefined scope of this follow-up study. We thus are unable to confirm the clinical significance of our findings, especially if DNR orders were honored in our cohort because the study reports a proxy outcome (documentation) rather than the true functional outcome (alignment of care delivery). Third, frailty was assessed clinically at baseline but through telephone interviews at follow-up. This change from clinician-based to self- or proxy-reported assessment may limit measurement comparability and introduce recall bias or misclassification, thereby reducing the reliability of conclusions regarding frailty progression. Fourth, multiple secondary outcomes were evaluated without adjustment for multiple comparisons, and the resulting analyses should therefore be interpreted as exploratory.

## CONCLUSION

Several years after a shared decision-making code status discussion, patients recalled little specific knowledge about resuscitation measures and their prognostic outcomes. However, the intervention group maintained a higher documented preference for DNR. These findings suggest that the decline in knowledge over time may represent a barrier to optimal long-term decision-making and highlight the need for repeated discussions to reinforce understanding and support informed, consistent preferences in this patient population.

## Data Availability

The data that support the findings of this study are available from the corresponding author upon reasonable request.

## Declarations

### Clinical Trial Number of the Original CLEAR Trial

NCT03872154; Registration Date/Update: 2024-03-08.
